# Methods to Assess the Impact of Mass Oral Cholera Vaccination Campaigns under Real Field Conditions

**DOI:** 10.1371/journal.pone.0088139

**Published:** 2014-02-07

**Authors:** Jacqueline Deen, Mohammad Ali, David Sack

**Affiliations:** 1 Menzies School of Health Research, Casuarina, Northern Territory, Australia; 2 Johns Hopkins Bloomberg School of Public Health, Baltimore, Maryland, United States of America; Public Health England, United Kingdom

## Abstract

There is increasing interest to use oral cholera vaccination as an additional strategy to water and sanitation interventions against endemic and epidemic cholera. There are two internationally-available and WHO-prequalified oral cholera vaccines: an inactivated vaccine containing killed whole-cells of *V. cholerae* O1 with recombinant cholera toxin B-subunit (WC/rBS) and a bivalent inactivated vaccine containing killed whole cells of *V. cholerae* O1 and *V. cholerae* O139 (BivWC). The efficacy, effectiveness, direct and indirect (herd) protection conferred by WC/rBS and BivWC are well established. Yet governments may need local evidence of vaccine impact to justify and scale-up mass oral cholera vaccination campaigns. We discuss various approaches to assess oral cholera vaccine protection, which may be useful to policymakers and public health workers considering deployment and evaluation of the vaccine.

## Introduction

Cholera continues to be a public health threat in many developing countries as highlighted by recent outbreaks in Angola, Zimbabwe, Haiti, the Democratic Republic of the Congo and other regions of Africa. Ensuring clean water, sanitation and hygiene constitute the main strategies for the prevention of the disease. But in endemic areas with seasonal cholera outbreaks, these basic needs are often not met and cholera outbreaks during natural or man-made disasters are usually associated with infrastructure breakdown. In October 2009, the World Health Organization’s Strategic Advisory Group of Experts on Immunization recommended that oral cholera vaccination should be considered as a reactive strategy during outbreaks, in addition to the already recommended preventive use of oral cholera vaccination in endemic areas [1]. The WHO has initiated the development of an oral cholera vaccine (OCV) stockpile, which will supply the vaccine in areas of emergency need [2].

Sinclair and co-workers have previously reviewed the safety, relative effectiveness and duration of protection of the different types of OCV [3]. Currently, there are two internationally-available and WHO-prequalified OCVs: an inactivated vaccine containing killed whole-cells of *V. cholerae* O1 with recombinant B-subunit of cholera toxin (WC/rBS) marketed as Dukoral™ and a bivalent inactivated vaccine containing killed whole cells of *V. cholerae* O1 and *V. cholerae* O139 (BivWC) marketed as Shanchol™. Both WC/rBS and BivWC are given in 2 doses. WC/rBS is taken with a bicarbonate buffer, which protects the B-subunit component from degradation by gastric acid but BivWC does not require a buffer.

Large-scale efficacy trials of earlier versions of WC vaccine with and without the B subunit in Matlab, Bangladesh showed that the vaccine is safe and provides 85% protection for 4–6 months after vaccination, 62% protection at 1 year, and 58% protection at 2 years [4]. Protection in children 6 years of age was 100% for the first 4–6 months but decreased rapidly thereafter. A clinical trial of the WC/rBS among military volunteers in Peru confirmed significant protective efficacy against cholera (86%) in the short term [5]. Post-licensure, observational studies of WC/rBS using a case-control approach in Mozambique [6] and a cohort design in Zanzibar [7] showed vaccine effectiveness of 78% and 79%, respectively, during the year after vaccination. In addition to providing direct protection to vaccine recipients, WC/rBS confers significant herd protection to neighboring non-vaccinated individuals [7], [8]. It has been deployed in various mass campaigns vaccinating those who are healthy, two years of age and older or not pregnant [9], but is expensive and used mainly by Western travellers. Aside from protection against cholera, WC/rBS (unlike BivWC) contains the B-subunit of cholera toxin, which cross-protects against heat-labile toxin producing *Escherichia coli* diarrhea for 3 to 5 months following vaccination [10], [11].

A large-scale efficacy trial of BivWC in India showed that the vaccine is safe and confers 67% protection against cholera within two years of vaccination [12], 66% at three years [13] and 65% at five-years [14] of follow-up. BivWC has also been shown to confer herd protection [15]. The vaccine has been used in various mass campaigns vaccinating those who are healthy, one year of age or older and not pregnant [9] but there are, as yet, no published studies of assessments of Shanchol effectiveness under real field conditions.

Despite available data on the protection conferred by OCVs, governments may require local evidence of impact to justify an initial vaccination campaign, for advocacy, to maintain public confidence in the vaccine or to guide future decisions regarding scaling-up of vaccination or inclusion of the vaccine in their public health program. This is an overview of epidemiologic methods that may be employed to assess the protection conferred by mass oral cholera vaccination campaigns and the requirements to carryout such assessments. To our knowledge, there have been only two published post-licensure observational studies that measured direct and indirect effectiveness of currently available OCVs [6], [7]. We drew on insights from these studies, along with general epidemiologic guidelines, experience with other vaccines and mathematical models, to provide a general overview of assessing the impact of OCVs. This paper is divided into two general sections: field studies to estimate OCV effectiveness and mathematical modeling to assess the potential impact of OCV. These approaches are summarized in Table 1.

**Table 1:** Methods to assess the impact of mass oral cholera vaccination campaigns.

	Comparison between	Outcome
**1. Field studies of effectiveness**		
**a. Main observational designs:**		
Case-control design	Proportion vaccinated among the cholera cases and proportion vaccinated among the healthy controls	Odds ratio of having received oral cholera vaccine among the cholera cases relative to healthy controls
Cohort design	Cholera incidence in the vaccinated and choleraincidence in the unvaccinated	Relative risk of cholera among the vaccinated relative to the unvaccinated cohorts
**b. Geographic Information System approach**	Cholera incidence in the non-vaccinated residents of neighborhoods with high vaccine coverage and cholera incidence in the non-vaccinated residents of neighborhoods with low vaccine coverage	Test for trend
**c. Before-and-after comparison**	Cholera incidence in the population before vaccination and cholera incidence in the same population after deployment of OCV	Percentage decline of cholera incidence or elimination
**2. Mathematical modeling**	Various vaccination strategies in the defined population and no vaccination in the defined population	Estimated reduction in cholera cases

## Analysis

### 1. Field studies to estimate OCV effectiveness

When estimating the protection conferred by mass oral cholera vaccination in a developing country setting, the distinction must be made between efficacy and effectiveness [16]. Vaccine efficacy is generally measured pre-licensure using a double-blind randomized controlled trial (RCT) study design under ideal research conditions. Vaccine effectiveness is the protection conferred when the vaccine is used under routine conditions in the community. Vaccine impact may have a broader connotation than vaccine effectiveness, including protection from cholera as well as reductions in cases and deaths. This section will focus on methods to assess the effectiveness of mass oral cholera vaccination campaigns in a developing country setting.

The effectiveness of OCV, measured at the population level, rests on vaccine efficacy and several factors. Decreased immunogenicity of the vaccine caused by, for example, the improper storage and transport of the vaccine or the inappropriate administration and timing of doses may lower effectiveness. Effectiveness may also be affected by immune response and other host factors of the population, location (e.g. endemic area with seasonal outbreaks versus natural disaster site with no previous cholera), cholera transmission intensity, as well as the timing and conditions under which the OCV mass vaccination is conducted (e.g. pre-emptive versus reactive campaign).

Mass OCV campaigns protect those who are vaccinated from cholera and result in a decrease in the number of individuals shedding *V. cholerae* in the community. This reduces the *V. cholerae* biomass in the immediate environment, lessening the likelihood of residents (vaccinated and unvaccinated) becoming infected. The protection conferred to the vaccinated individuals due to vaccine-induced immune response is direct protection, whereas that conferred to the non-vaccinated is indirect (herd) protection. Considering the large indirect effect of OCV [7], [8], [15] and given the importance that this herd effect can have on assessing outcome, anyone interested in an OCV campaign should ideally plan their intervention and assessment of protection in a manner that takes herd immunity into account. Furthermore, the use of OCV in programs to control endemic cholera has been found to be cost-effective when herd effect is included in the calculations [17].

Several studies have demonstrated the direct and indirect protection conferred by BivWC and WC/rBS [3]–[9], [12]–[15] in various settings. Conducting further RCTs would be expensive and require ethical justification for withholding the vaccine from a control group. When such study designs cannot be used to obtain local data, observational study designs have to be employed but careful attention has to be paid to reliability issues, including bias, misclassification and confounding. The absence of randomly selected control groups in these observational studies render such designs vulnerable to bias. Individuals included in these observational studies decide whether or not to take the vaccine and also decide whether or not to seek treatment from a health care provider if he or she develops acute watery diarrhea. (The individual “decisions” may relate to access to the vaccine program as well as access to the treatment options). These inherent differences in characteristics and health-seeking behavior between the vaccinated and unvaccinated groups can bias measurements of OCV effectiveness and are the main limitation of measuring vaccine effectiveness using a non-randomized design. For example, those who choose not to participate in an OCV campaign may also not seek treatment for acute, watery diarrhea, resulting in a lower detection of cholera cases among the non-vaccinated and thereby falsely reducing the effectiveness estimate. Any factor that differentially raises or lowers the detection of cholera cases in either the vaccinated or unvaccinated group will bias the OCV effectiveness estimate. Since patients with diarrhoea are usually unaware of whether they have cholera or not, any factor that differentially raises or lowers the detection of cholera cases will likely do the same to non-cholera cases. Recent observational studies assessing OCV effectiveness [6], [7] have incorporated concurrently conducted “bias-indicator” studies of non-cholera diarrhoea (discussed below). Such parallel studies can detect but not remedy bias due to differential diarrhoea care-seeking behaviour. The aim of the parallel study is to evaluate whether there is unexpected protection from OCV against non-cholera diarrhoea, resulting from differential seeking of medical care for diarrhoea among those who chose to receive the vaccine and those who did not. Using non-cholera diarrhoea as a bias-indicator should only be done if the stool culture methods to confirm cholera are rigorously checked (for example, by quality assurance testing and cross checking of samples in a reference laboratory) as poor stool culture techniques resulting in low sensitivity may result in misclassification of cholera as non-cholera cases.

There is also the problem of potential confounding in observational studies. Potential confounders in OCV effectiveness assessments include age, sex, socio-economic status, educational level, sanitation, water supply and distance to a vaccination center and treatment facility. These factors may be associated with both participation in the vaccination campaign and with the risk for or the detection of cholera. In RCT studies, confounding is avoided by random distribution of individuals between the vaccinated and unvaccinated groups. In observational studies, potential confounders have to be considered and controlled for by other means. Confounding can be adjusted for in the design (through matching) or during the analysis (through stratification or multi-variable analysis). For example, in case-control studies, matching for age, sex and neighborhood may help ensure comparability between cases and controls. During the analysis, a comparison of characteristics between cases and controls (in case-control studies) and between the vaccinated and unvaccinated individuals in the population (in cohort studies) may provide an indication of comparability; statistical methods may be used to adjust for these confounders. Methods to control confounding in the analysis can range from simple stratification to multivariate regression to produce adjusted effectiveness results.

There are several basic components that are essential for the assessment of both direct and indirect OCV effectiveness following a mass vaccination campaign, which are shown as a checklist in Figure S1. The first essential step is the development of standard operating procedures for the OCV campaign. A detailed discussion of the planning and logistics of mass OCV campaigns are available from WHO [18]. The validity of the OCV protection estimates will depend on how well the components are implemented including the completeness of case capture from the target population, the uniform application of the case definition and the ascertainment of vaccination status. Importantly, field studies to assess OCV protection can only be carried out if there are a sufficient number of cases following the vaccination campaign. OCV campaigns with very high coverage may interrupt cholera transmission resulting in the absence of cases. The timing of the vaccination campaign is also crucial in the assessment of effectiveness. The outbreak curve of cholera cases may already be on the downward slope or the outbreak may be ending when the vaccination campaign is carried out.

#### a. Observational study designs to estimate OCV direct protection

There are different study designs [19]–[21] that may be used to assess OCV direct protection following a mass vaccination campaign. Case-control and cohort designs were selected as the main approaches based on their applicability in assessing OCV effectiveness, as well as previous experience with their use.

In a case-control design, cholera cases are compared with controls who are purposefully selected and comparable to the cases except for not having diarrhea during the focal time. The focal time is the period from the mass vaccination until the case develops cholera. Cases and controls should be from the defined population (also known as source population). Controls may be matched to each case for variables known to be associated with the exposure and outcome (e.g. age, sex and neighborhood) or unmatched and confounders adjusted for during the analysis. Matching of cases and controls may increase the likelihood that the detected outcome (cholera or no cholera) is due to differences in the exposure (vaccination or no vaccination) rather than due to confounders such as age, sex and neighborhood. But matching complicates the analysis and makes it impossible to evaluate the impact of the factor on which matching is done.

In a previous study [6], aside from matching for the above characteristics and to further ensure similar risk for cholera, controls were selected from the neighborhood of the case using a standard procedure (i.e. controls were chosen through a walk around procedure starting from every third house to the right of the case’s house). Coming from the same neighborhood was considered as a proxy for matching of socio-economic status, sanitation and water supply. Generally, 2 to 4 controls per case are included; there is no practical increase in study power when more than 4 controls per case are included [22]. Instead of neighborhood controls, using hospital controls (for example confirmed shigella cases) would match cholera cases in terms of health care utilization. However in developing country settings where cholera occurs and where these studies are to take place, there is often limited facility for diagnosing the cause or causes of a diarrheal illness; laboratory confirmation of cholera will be difficult enough.

In a case-control design, OCV effectiveness is calculated by comparing the odds ratio (OR) of vaccination between the cases and controls [19]. The basic formula for calculating the OR and vaccine effectiveness are shown in Table 2. In the study in Beira, Mozambique, there were 43 cholera cases from the defined population during one year following the mass vaccination [6]. 172 matched healthy controls (4 per case) were included in the study. 10/43 (23%) of the cholera cases and 94/172 (55%) of the matched controls received at least one dose of OCV resulting in an unadjusted OR of 0.19. The OR, adjusted for differing characteristics between cases and controls, was 0.22 and the calculated vaccine effectiveness within one year following vaccination was 78% (i.e. [1–0.22] * 100%).

**Table 2:** Formula for calculating odds ratio (OR) and vaccine effectiveness in studies using a case-control design.

	Cases	Controls
Vaccinated	a	B
Unvaccinated	c	D
	OR = ad/bc	

Vaccine effectiveness (%) = (1–OR)×100.

A cohort study to assess OCV effectiveness may be appropriate when a defined population can be followed prospectively after a mass vaccination campaign. This study design is considered as quasi-experimental, that is, OCV is not randomly allocated but nonetheless a study is conducted of the subjects grouped according to whether they were or were not vaccinated. Vaccine effectiveness is measured using the incidence of cholera among the vaccinated and unvaccinated persons in the population. The basic formula is: vaccine effectiveness (%) = (Incidence in unvaccinated–Incidence in vaccinated/Incidence in unvaccinated or relative risk)×100 [19].

It is important to ensure that efforts to detect cases among unvaccinated and vaccinated persons are equal. As discussed above, presentation to health centers may be associated with previous participation or non-participation in the mass vaccination campaign. There may also be an unequal chance of exposure to cholera between vaccine recipients and non-recipients; a comparison of characteristics between vaccine recipients and non-recipients may provide an indication of the comparability of both groups and may be used to adjust the statistical calculations.

In the study in Zanzibar, 23,921 (50%), 3,757 (8%) and 20,500 (42%) of the target population of 48,178, received two, one and zero OCV doses, respectively, during the mass vaccination [7]. Vaccine recipients differed in several aspects from those who did not receive the vaccine. Vaccine recipients were more likely to be female and younger that non-recipients. Vaccine recipients were less likely to drink tap water, more likely to have had a recent history of diarrhea, to live in less densely populated areas and to live in areas with higher neighborhood-level vaccine coverage. 42 cholera cases were detected within 14 months after the mass vaccination campaign. Six of 23,921 recipients of two vaccine doses had cholera (incidence of 0.25 per 1000 persons) compared with 33 of 20,500 unvaccinated people (incidence of 1.61 per 1000 persons). The RR, adjusted for significantly different variables between the vaccinated and unvaccinated groups was 0.21. Receipt of two complete doses of vaccine resulted in protective effectiveness of 79% (i.e. [1–0.21] * 100%).

To check for possible bias resulting from the differential seeking of medical care among those who received the vaccine and those who did not, a bias-indicator study may be conducted in parallel with the primary cholera study [6], [7]. For a case-control design, aside from the primary cholera assessment, a concomitant study may be simultaneously done wherein non-cholera diarrhoea cases are matched with healthy controls and vaccine effectiveness against non-cholera diarrhoea calculated. For cohort studies, the incidence of non-cholera diarrhoea among the vaccinated and unvaccinated persons in the study population may be used to calculate vaccine effectiveness against non-cholera diarrhoea. The lack of OCV protection against non-cholera diarrhea would suggest the absence of bias due to the differential seeking of medical care among the vaccinated and unvaccinated. SInce WC/rBS provides short-term protection against heat-labile toxin producing *E. coli* diarrhea [10], [11], bias-indicator studies incorporated into assessments of WC/rBS effectiveness should start 3 to 5 months after the mass vaccination to exclude patients who are cross-protected against enterotoxigenic *E. coli*.

In the Beira study, a concurrently conducted bias-indicator assessment comparing persons with noncholeraic diarrhea and healthy controls in the same population found no protection associated with receipt of the vaccine, which was interpreted as the absence of bias in the primary study [6]. In the Zanzibar study, the potential for bias was also investigated by assessing whether the vaccine protects against non-cholera diarrhea [7]. Surprisingly, not only did vaccination not protect against non-cholera diarrhea as expected, but it was associated with a higher risk of non-cholera diarrhea. This was interpreted as an absence of bias and that people who were at high risk for diarrhea were more likely to participate in the vaccination campaign than those who were not.

Other study designs may be considered. For example, a vaccine probe approach may be used. Unlike studies of vaccine efficacy or effectiveness that assess proportionate disease reduction between the vaccinated and unvaccinated, vaccine probe studies measure absolute disease reduction, known as the “vaccine preventable disease incidence” [23]. The distinction is important as vaccines may have an impact on disease severity but not on incidence. Vaccine probe studies are particularly useful when laboratory diagnosis is difficult (e.g. pneumococcal and *Haemophilus influenzae* type b disease) or when the infection may lead to complications at which time etiologic confirmation may not be possible (e.g. influenza leading to secondary bacterial pneumonia) [23]. Ideally vaccine probe studies should involve randomization and blinding, which may be difficult to implement for licensed vaccines such as OCV.

Alternative approaches such as case-cohort designs, interrupted time series analysis or case-control studies using different control groups may also be considered. There is as yet no experience with the use of these designs in the evaluation of OCV effectiveness.

#### b. Geographic Information System (GIS) approach to estimate OCV indirect protection

A straightforward approach to measure herd immunity is to conduct cluster randomization trials, in which groups of individuals serve as the units randomised to the vaccine and control arms of the trial [24]. Indirect vaccine protection is measured by comparing the disease incidence in the non-vaccinated members of clusters assigned to receive the vaccine versus those in clusters receiving placebo. However, during post-licensure use of OCV in mass vaccination campaigns, the distribution of vaccine is not randomised; innovative observational methods have to be utilized to assess herd protection.

Geographic Information System (GIS) mapping may be utilized to assess indirect protection conferred by OCV deployed during mass vaccination campaigns. The GIS method takes advantage of varying levels of vaccine uptake within neighbourhood contacts of the individual that may occur due to chance or differing rates of participation. To evaluate indirect protection, cholera incidence among non-vaccinated residents of neighborhoods with high vaccine coverage is compared with cholera incidence among non-vaccinated residents of neighborhoods with low vaccine coverage (Table 1).

Aside from the components of OCV effectiveness assessment discussed above and in Figure S1, there are additional requirements to measure indirect protection using the GIS approach. First, household mapping of the target population is required to carryout this analysis. A census database of the target population is essential and this will be correlated to a map of the area using GIS. The correlation is generally done using a ground survey to link each household with a geographic point on the map. The census database is also correlated with the vaccination database such that participants and non-participants of the mass vaccination campaign can be linked to a household geographic location. Cholera cases identified during surveillance will also be linked to a household geographic location.

**Figure 1:**
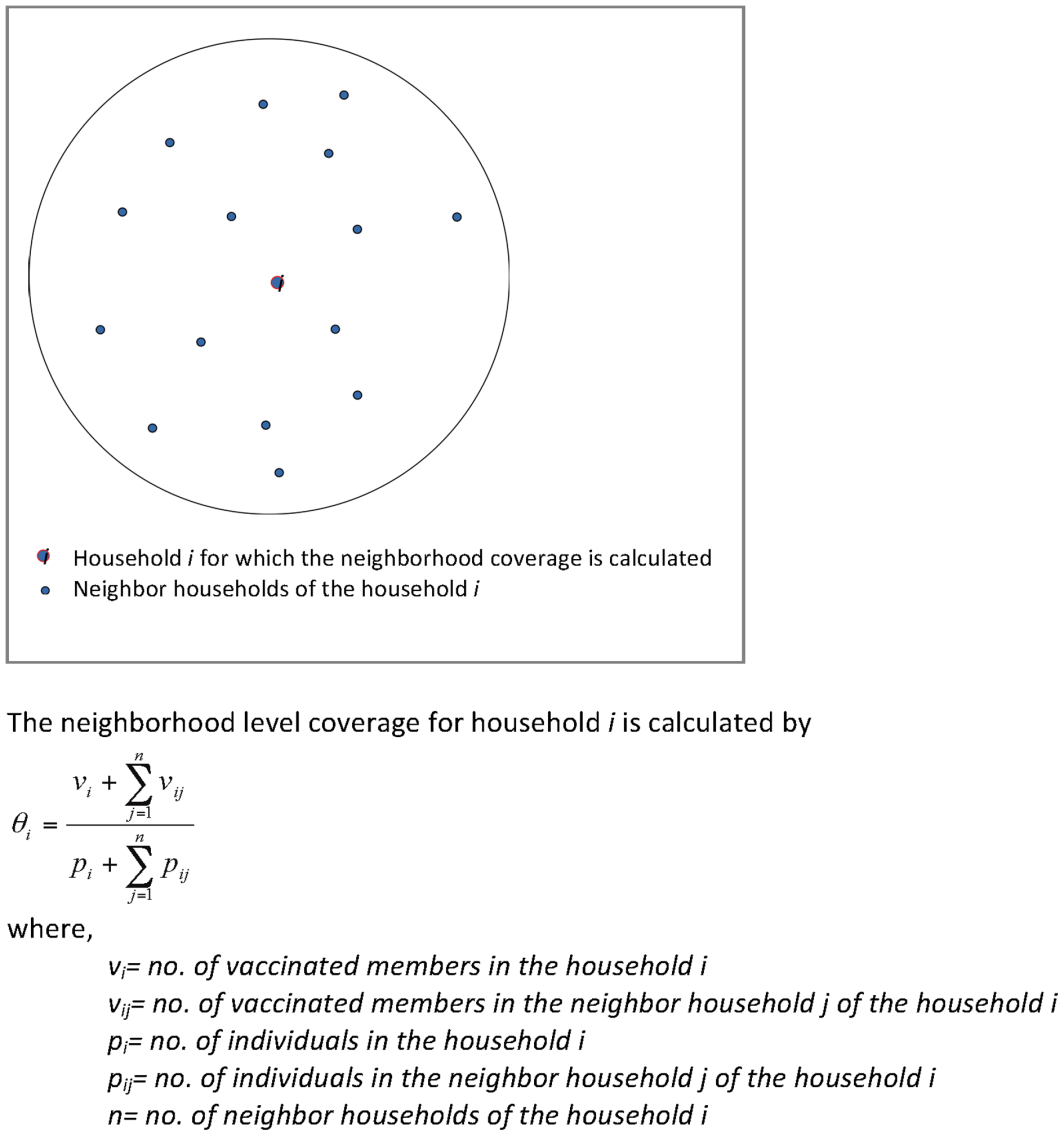
Computation of neighborhood level vaccine coverage using a Geographic Information System approach.

Next, it is important to define an appropriate (optimal) size of neighborhood for computing neighborhood-level vaccine coverage for each household in order to evaluate geographic variation of vaccine coverage within the study area. The premise behind computing neighbourhood-level vaccine coverage is that individuals are more likely to interact with those who are close to them in space than those located further away [25]. Thus, disease rates within each neighbourhood represent exposure from an individual’s contact network that leads to the transmission of cholera [26]–[28]. The probability of becoming infected within each neighbourhood depends on the number of infected and vaccinated individuals within the contact network. Different neighborhood sizes need to be investigated to find the optimal neighborhood size. The underlying assumption for defining an optimal neighborhood size is that it should not be too small as to yield an unstable result and not too big such that local detail is obscured. Different neighborhood sizes can be tested for suitability using Hartley’s variance ratio test [29], as has been done elsewhere [7], [8]. Computation of the neighborhood level vaccine coverage using the GIS approach is shown in Figure 1 [30]. Contour mapping techniques (ESRI, Redlands, CA, USA) may be used to show spatial patterns of neighborhood-level vaccine coverage in the study area, as has been previously done [7], [8]. If multiple neighbourhood sizes are used, there is a risk of false positives and the p-value needs to be corrected for multiple comparisons [31].

In mass vaccination campaigns, the neighborhood level vaccine coverage is defined *post hoc*. Therefore, the approach can be affected by many non-random factors affecting neighborhood level vaccine coverage [7], [8]. Care should be taken to adjust the analyses for factors that might bias the association between levels of vaccine coverage and the disease rates. And as with case-control and cohort designs, a parallel bias-indicator GIS study may also be incorporated in the assessment.

GIS methods employed in the Zanzibar study [7] showed that cholera cases were mainly located outside the high-level vaccine coverage areas. In contrast, non-cholera cases were randomly distributed. There was a decrease in the incidence of cholera among the non-vaccinated residents within a neighborhood as the vaccine coverage in that neighborhood increased. Such an inverse relation was not noted for non-cholera diarrhea (bias-indicator study). A comparison of the risk for cholera in unvaccinated residents between the highest and lowest neighborhood level vaccine coverage showed a 75% indirect protection in the higher coverage group compared with the lower coverage group.

#### c. Before-and-after comparison

When OCV is deployed on a large-scale in an endemic area, particularly with the aim of disease elimination, the annual incidence of cholera during the period before and after deployment of the vaccine could be compared to show vaccine impact, as has been done for other vaccines [32], [33]. If the annual number of cases is few, then a cumulative incidence over a several year period may be utilized. A before-and-after comparison would require continuing surveillance for cholera prior to, during and following vaccination. An observed reduction or elimination of cholera would suggest that OCV reduced or interrupted *V. cholerae* transmission (likely through the combination of direct and indirect protection). However, a cause-and-effect relationship would be difficult to establish since cholera outbreaks are unpredictable even over a multi year time horizon and other factors such as improvements of water supply and sanitation, changes in socio-economic status or environmental factors affecting *V. cholerae* ecology [34] may decrease disease transmission. Nevertheless, this method may be useful to bolster confidence in OCV and as an indicator of when and where repeat OCV campaigns may be necessary. Herd effects may also be apparent in non-vaccinated age groups within the same community and may be used assess indirect protection, as has been done in other vaccination programs [35]. It would be interesting to find out whether wide-scale mass OCV vaccination in a relatively closed cholera-endemic environment (e.g. an island community) without changes in water and sanitation infrastructure could eliminate cholera.

### 2. Mathematical modeling to assess the potential impact of OCV

Stochastic simulation models have been used to investigate the potential control of endemic cholera using OCV. After the 1985 vaccine trial results from Matlab, Bangladesh [4] was reanalyzed using the GIS approach to show herd protection from OCV [8], Longini and co-workers used the same dataset to construct a simulation model of varying vaccine coverage levels and cholera illness [36]. They calculated that 50% OCV coverage in this population would result in 89% reduction in cholera cases even among the unvaccinated, and a 93% reduction overall in the entire population. Their estimates would apply only where cholera is endemic and population levels of immunity (from previous exposure to the disease) are relatively high.

More recently, following the 2010–2011 cholera outbreak in Haiti, various simulations were used to assess different vaccination strategies [37]. The overall effectiveness of a vaccination strategy was estimated as the percentage of cases averted with respect to the baseline simulations in which vaccines were not used. The most efficient reactive strategy found was to prioritize vaccination of individuals living in cholera high-risk areas. In Haiti, the population living along the lower Artibonite River was considered as having a higher exposure to cholera and greater potential to transmit the disease. The modeling showed that targeting one million doses of vaccine to these areas, enough for two-dose vaccination of 5% of the population, would decrease the number of cases by 11%. The same strategy with enough vaccine for 30% of the population with modest hygienic improvement could reduce cases by 55% and save 3,320 lives.

Mathematical modeling is a valuable method to study cholera outbreaks and to simulate the effect of different vaccination strategies and other interventions. These calculations are useful to determine the most optimal vaccination strategy (particularly when there are an insufficient number of vaccine doses available) and to guide local vaccination programmes and donor assistance. But these estimates are only as good as the assumptions that are used to calibrate the models [38]. There remains the need for field studies to validate these approximations.

## Discussion

In summary, we discussed various post-licensure approaches to assess OCV protection. The study design chosen to measure OCV effectiveness will depend on logistics and resources, e.g. whether it is possible to recruit controls from the target population for a case-control study or whether the target population can be followed longitudinally for a cohort study. Because of the non-randomized nature of these studies, there is a potential for bias and confounding in these assessments and they should incorporate procedures to assess and control for these. Evaluation of protection may be done if there is incomplete OCV coverage of the target population and if there are cholera cases following the mass vaccination. It would not be possible to assess OCV protection when there are few or no cholera cases detected after a campaign except through before-and-after comparisons. The outcome from these assessments should be interpreted with caution, keeping in mind study limitations and should consider previous evidence. Mathematical modelling may also be used to assess the impact of various vaccination strategies tailored to different outbreak scenarios.

This paper is limited because it does not provide detailed information on methodology. On the other hand, the article brings together information from various studies and provides a general overview of the methods to assess the impact of OCVs. Donor and implementing agencies, as well as in Ministries of Health staff in many countries where cholera is endemic, may not be fully aware of the methods that are available to assess the impact of OCVs. Compared with other vaccines given in mass campaigns, OCVs are underutilized and country-specific studies of protection may be useful to inform policy decisions and provide additional support for more widespread use of OCVs in areas affected by cholera. There is a need for detailed guidelines on assessing the impact of OCV that include protocol and case report form templates, such as those available for other vaccines [39], [40].

## Supporting Information

Figure S1 **Checklist of the main requirements for an OCV effectiveness assessment.**
(TIFF)
